# Soil Application of Selenium in Wheat (*Triticum aestivum* L.) Under Water Stress Improves Grain Quality and Reduces Production Losses

**DOI:** 10.3390/plants13243460

**Published:** 2024-12-10

**Authors:** Leônidas Canuto dos Santos, Gabryel Silva Martins, Pedro Antônio Namorato Benevenute, Jucelino de Sousa Lima, Fernanda Ribeiro dos Santos, Otávio Vitor Souza Andrade, Indira Pereira de Oliveira, Fábio Henrique Alves Bispo, Lívia Botelho, Flávio Henrique Silveira Rabêlo, Paulo Eduardo Ribeiro Marchiori, Luiz Roberto Guimarães Guilherme, Guilherme Lopes

**Affiliations:** 1 Department of Soil Science, School of Agricultural Science, Federal University of Lavras (UFLA), Lavras 37200-900, MG, Brazil; leonidas.santos2@estudante.ufla.br (L.C.d.S.); gabryel.martins2@estudante.ufla.br (G.S.M.); benevenutepedro@gmail.com (P.A.N.B.); fernanda.santos10@estudante.ufla.br (F.R.d.S.); fabiobispo2@gmail.com (F.H.A.B.); livia.botelho@ufla.br (L.B.); guilherm@ufla.br (L.R.G.G.); 2 Department of Biology, Institute of Natural Sciences, Federal University of Lavras (UFLA), Lavras 37200-900, MG, Brazil; sousajucelino12@gmail.com (J.d.S.L.); otaviovsandrade@gmail.com (O.V.S.A.); paulo.marchiori@ufla.br (P.E.R.M.); 3 Department of Agricultures, School of Agricultural Science, Federal University of Lavras (UFLA), Lavras 37200-900, MG, Brazil; indira.oliveira1@estudante.ufla.br; 4 Department of Soil Science, Luiz de Queiroz College of Agriculture (ESALQ), University of São Paulo, Piracicaba 13418-900, SP, Brazil; flaviohsr.agro@usp.br

**Keywords:** sodium selenate, water stress, agronomic biofortification, food security, tolerance mechanisms

## Abstract

Selenium (Se) is an essential element for humans. However, much of the world’s human population is deficient in this element, which has become a public health problem. This study aimed to evaluate whether applying severe water stress to wheat plants (*Triticum aestivum* L.) could allow Se to reduce the production losses and increase the grain quality, thereby contributing to the reduction in hidden hunger. The experiment was conducted in a randomized block design with four replications in a 5 × 2 factorial scheme, with five doses of Se (0.00, 0.25, 0.50, 1.00, and 2.00 mg dm^−3^) and two irrigation conditions (with and without water deficit). When sodium selenate (Na_2_SeO_4_) was applied to the soil, the grains were rich in Se. Under low doses, there was an enrichment of the grains in sulfur, iron, copper, and zinc as well as total free amino acids and total soluble proteins, and lower losses in productivity under severe water stress. Higher doses decreased the concentration of malondialdehyde (MDA) and hydrogen peroxide (H_2_O_2_), increased the catalase activity, and increased the water use efficiency (WUE). Therefore, applying Se at a dose of 0.25 mg dm^−3^ is effective for the biofortification of wheat grains. It enhances grain nutritional quality, increases Se bioaccessibility, and reduces production losses under water stress conditions.

## 1. Introduction

The well-being of the human species is centered on some pillars, among which food security can be considered the foundation, being one of the central components that deal with the Millennium Development Goals (MDG) of the United Nations (UN) [1]. Conceptually, food security will be efficient from the moment that all people, at all times, have social, physical, and economic access to sufficiently nutritious foods that meet their dietary needs for a healthy and active life [2]. However, another phenomenon, the so-called “hidden hunger,” has been observed, given by the deficiency of essential elements, vitamins, and in some cases, fatty acids and amino acids [3]. This situation is called hidden because micronutrient deficiency remains hidden until clinical symptoms are detected [4], which is a significant food security and public health problem.

Wheat (*Triticum aestivum* L.) is one of the oldest crops and has been widely cultivated by humans throughout history. Playing a vital role in the food and culture of many countries around the world, with about 217 million hectares planted annually, it is a food present in the diet of the majority of the world’s population, covering consumption by different ethnic and social classes, and is responsible for providing about one-fifth of the calories and proteins consumed by humans [5].

Specific vitamins and minerals are essential for the proper functioning and metabolism of the human body; among the micronutrients, Se is included, as it plays a role in several metabolic processes in the human body. Se stimulates immune system cells, particularly cytotoxic T cells, helper T cells, and natural killer (NK) cells [6]. On the other hand, deficiency in this essential nutrient is associated with hypothyroidism, muscular necrosis, male infertility, Keshan disease, Kashin–Beck disease, and cardiovascular and cerebrovascular diseases [7,8,9].

In addition to Se, an element beneficial to plants and essential to humans and animals, other trace elements, which are plant nutrients such as copper (Cu), iron (Fe), manganese (Mn), and zinc (Zn), may also be present in wheat grains and play important roles in the growth and development of plants and animals [10]. This consequently affects the nutritional quality of the food produced from this wheat and the health of the final consumers.

In a similar way to what occurs in the performance of Se in human health, this element can also contribute to an improvement in plant health, more specifically by acting in the fight against biotic and abiotic stresses; among the latter highlights whether in this work severe water stress was caused by lack of water. Scientific research has shown that Se can have significant impacts on drought-stressed plants. However, these impacts may vary depending on Se concentration, the specific plant conditions, and the degree of water stress [11,12,13].

The literature reports that under water deficit conditions, Se acts on plant protection, increasing the activity of antioxidant enzymes, helping to neutralize free radicals, and reducing oxidative stress in plants stressed by drought [14]. The presence of Se can improve the efficiency of photosynthesis, which is crucial since drought often impairs the photosynthetic capacity of plants [15]. In short, the exogenous application of Se can increase the plants’ ability to resist the harmful effects of water stress.

Wheat as well as other crops is continuously exposed to water stress. This condition is one of the most limiting environmental factors to plant growth and productivity, possibly causing significant famine [16]. This will threaten global food security in the future in the face of ever-changing scenarios and climate uncertainties, the depletion of water resources, and increased demand for food due to an alarming population growth [5,17,18]. In addition, understanding and promoting sustainable agricultural practices in wheat production is essential to ensure food security and promote balanced nutrition.

Therefore, the novelty of this research lies in investigating the optimal dose of Se applied via soil to mitigate the effects of severe water stress in wheat plants while also improving the nutritional quality of the grains. This study reveals the dual role of Se: in addition to biofortifying wheat grains by enriching them with essential elements for humans, it also improves the water use efficiency (WUE), antioxidant enzyme activity, and osmolyte levels, allowing the plants to reduce productivity losses under stress conditions. Furthermore, the research highlights the role of Se in increasing nutrient bioaccessibility, a critical factor for improving the absorption of essential elements by humans and enhancing food security, thus significantly advancing the understanding of Se’s benefits for wheat cultivation and stress mitigation. We hypothesize that applying endogenous Se via soil in wheat plants exposed to severe water deficit conditions protects the plants from water stress and enhances food security by improving nutrient bioaccessibility in a simulated human gastrointestinal system (in vitro).

## 2. Results

### 2.1. Grain Productivity and Mass

**Figure 1:**
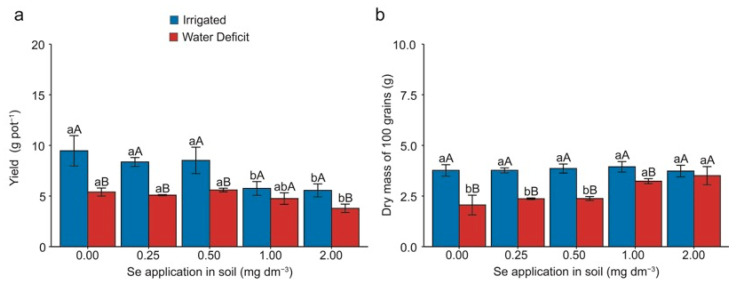
Effect of Se application on the grain yield (**a**) and dry mass of 100 grains (**b**) in wheat (*Triticum aestivum* L.) plants with and without water deficit. Lowercase letters compare the means among Se doses within each irrigation condition, and capital letters compare irrigation conditions within each Se dose. Equal letters did not differ statistically by the Duncan test (*p* < 0.05). The error bar represents the standard error of the means (n = 4).

The yield and dry mass of 100 grains were significantly influenced (*p* < 0.05) by both water deficit and applied Se doses (Figure 1). Under optimal irrigation, applying 1.00 and 2.00 mg Se dm^−3^ resulted in a mean decrease of 35% in productivity compared with doses of 0.00, 0.25, and 0.50 mg Se dm^−3^ (Figure 1a). When considering only plants subjected to water deficit, a mean decrease of 29% was observed for the application of 2.00 mg Se dm^−3^ compared with doses of 0.00, 0.25, and 0.50 mg Se dm^−3^. Comparing plants under irrigation with those cultivated under water deficit, it was noticeable that water deficit led to decreases of 43%, 39%, 34%, and 31% for doses of 0.00, 0.25, 0.50, and 2.00 mg Se dm^−3^, respectively. It is worth noting that there was no significant difference between the irrigated plants and those subjected to water deficit for the dose of 1.00 mg Se dm^−3^.

Regarding the dry mass of 100 grains, the application of 1.00 and 2.00 mg Se dm^−3^ resulted in a mean increase of 48% compared with doses of 0.00, 0.25, and 0.50 mg Se dm^−3^ under water deficit (Figure 1b). When comparing the condition of suitable irrigation and water deficit within each Se dose, it was observed that water deficit led to decreases of 45%, 37%, 38%, and 18% in doses of 0.00, 0.25, 0.50, and 1.00 mg Se dm^−3^, respectively. Notably, there was no significant difference between the irrigated plants and those subjected to water deficit for the 2.00 mg Se dm^−3^ dose.

### 2.2. Gas Exchange

**Figure 2:**
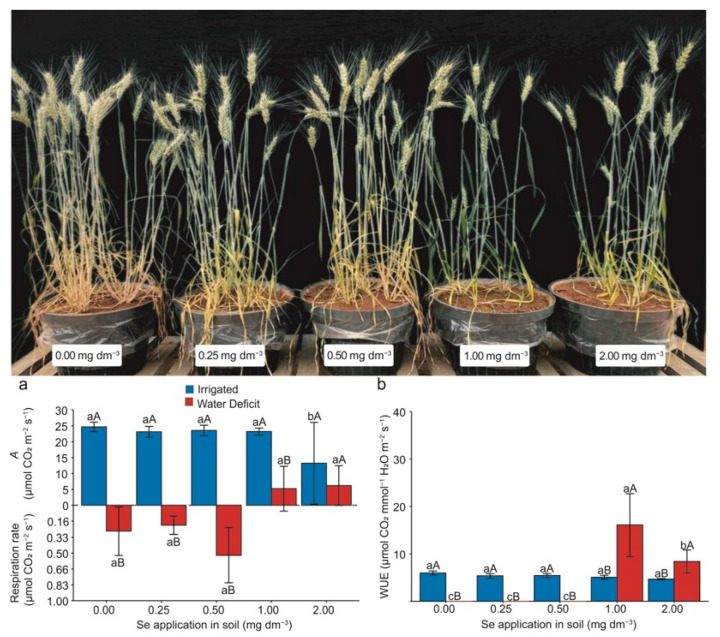
Visualization of water stress in wheat (*Triticum aestivum* L.) plants and the effect of Se application on the photosynthetic rate—*A* (**a**) and water use efficiency—WUE (**b**). Lowercase letters compare the means among Se doses within each irrigation condition, and capital letters compare the irrigation conditions within each Se dose. Equal letters did not differ statistically by the Duncan test (*p* < 0.05). The error bar represents the standard error of the means (n = 4).

The photosynthetic rate (*A*) and WUE were significantly affected (*p* < 0.05) by water deficit and the applied Se doses during stress (Figure 2). For *A*, a 100% decrease, turning negative, was observed for the 0.00, 0.25, and 0.50 mg Se dm^−3^ doses when plants were subjected to water deficit, indicating respiration. In contrast, the 1.00 and 2.00 mg Se dm^−3^ doses experienced a smaller decrease (−75%) (Figure 2a). Notably, the 1.00 and 2.00 mg Se dm^−3^ doses promoted an increase of approximately 521% compared with the other doses under water deficit. For the WUE, drastic reductions were observed for the 0.00, 0.25, and 0.50 mg Se dm^−3^ doses. However, the 1.00 and 2.00 mg Se dm^−3^ doses led to average increases of 203% and 58%, respectively, compared to the irrigated plants treated with the same doses (Figure 2b).

### 2.3. Enzymatic Antioxidant Metabolism and Oxidative Damage

**Figure 3:**
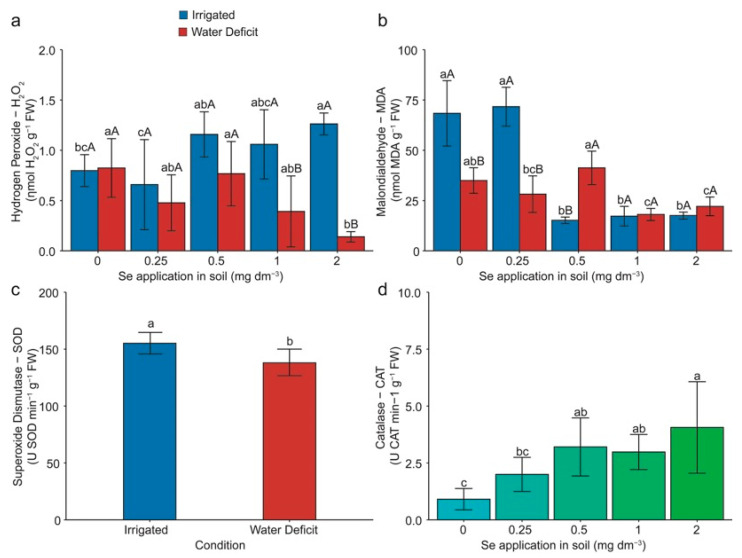
Effect of Se application and water deficit on hydrogen peroxide (**a**), malondialdehyde (**b**), superoxide dismutase (**c**), and catalase (**d**) in wheat (*Triticum aestivum* L.) plants. For the variables superoxide dismutase (**c**) and catalase (**d**), there was no significant interaction between the factors (*p* > 0.05), indicating that the effects of each factor are independent. Lowercase letters compare the means among Se doses within each irrigation condition, and capital letters compare the irrigation conditions within each Se dose. Equal letters did not differ statistically by the Duncan test (*p* < 0.05). The error bar represents the standard error of the means (n = 4).

The contents of hydrogen peroxide (H_2_O_2_) and malondialdehyde (MDA) were significantly influenced (*p* < 0.05) by both the water deficit and applied Se doses. In contrast, the antioxidant enzymatic activity of superoxide dismutase (SOD, EC 1.15.1.1) was influenced only by the water deficit, and catalase (CAT, EC 1.11.1.6) only by the applied Se doses (Figure 3).

Regarding the H_2_O_2_ content, it was noted that the dose of 2.00 mg Se dm^−3^ led to a 58% increase compared with 0.00 mg Se dm^−3^ under the optimal irrigation conditions (Figure 3a). Additionally, doses of 0.50 and 2.00 mg Se dm^−3^ resulted in a mean increase of 94% compared with the application of 0.25 mg Se dm^−3^ under irrigation. Under water deficit conditions, the application of 2.00 mg Se dm^−3^ decreased by approximately 81% compared with the 0.00 and 0.50 mg Se dm^−3^ doses. Reductions of 62% and 88% were observed for applications of 1.00 and 2.00 mg Se dm^−3^, respectively, when compared with the optimal irrigation conditions and water deficit within each dose.

Regarding MDA, a mean increase of 320% was observed for doses of 0.00 and 0.25 mg Se dm^−3^ compared with the others under the optimal irrigation conditions (Figure 3b). Under the water deficit, applying 1.00 and 2.00 mg Se dm^−3^ resulted in mean decreases of 42%, 28%, and 50% compared with doses of 0.00, 0.25, and 0.50 mg Se dm^−3^, respectively. Additionally, it was found that the dose of 0.50 mg Se dm^−3^ led to an increase of 47% compared with 0.25 mg Se dm^−3^ under water deficit. When comparing the irrigated and water deficit conditions, a decrease in MDA content of 50% and 60% was observed for doses of 0.00 and 0.25 mg Se dm^−3^, respectively, and an increase of 170% for the dose of 0.50 mg Se dm^−3^ (Figure 3b).

SOD activity was reduced by 11% when comparing the irrigated plants with those under water deficit (Figure 3c). Regarding the CAT activity, the application of 2.00 mg Se dm^−3^ increased by ~180% compared with doses of 0.00 and 0.25 mg Se dm^−3^, while the application of 0.50 and 1.00 mg Se dm^−3^ led to a mean increase of 240% compared with plants without Se application (Figure 3d).

The proline content was significantly affected (*p* < 0.05) by both the water deficit and Se doses (Table 1). Under adequate irrigation, the application of 0.25 and 0.50 mg Se dm^−3^ resulted in a mean increase of 35% compared with doses of 0.00 and 1.00 mg Se dm^−3^ and 295% compared with the dose of 2.00 mg Se dm^−3^. Additionally, there was a mean increase of 191% for doses from 0.00 to 1.00 mg Se dm^−3^ compared with the application of 2.00 mg Se dm^−3^. Adding 0.25 to 0.50 mg Se dm^−3^ for plants subjected to water deficit resulted in a mean increase of 56%, 123%, and 368% compared with doses of 0.00, 1.00, and 2.00 mg Se dm^−3^, respectively. When comparing the irrigated and water deficit conditions within each dose, a 28% increase was observed at 0.50 mg Se dm^−3^ under water deficit, and a 36% decrease at 1.00 mg Se dm^−3^.

### 2.4. Grain Quality and Bioaccessibility in the Simulated Human Gastrointestinal System

**Figure 4:**
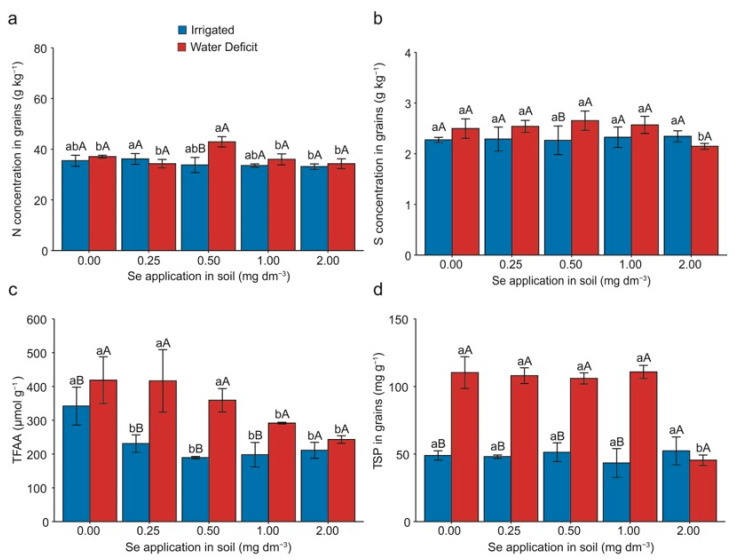
Effect of Se application and water deficit in nitrogen—N concentration (**a**), sulfur—S concentration (**b**), total free amino acids—TFAAs (**c**), and total soluble proteins—TSPs (**d**) in wheat (*Triticum aestivum* L.) plants. Lowercase letters compare the means among Se doses within each irrigation condition, and capital letters compare the irrigation conditions within each Se dose. Equal letters do not differ statistically by the Duncan test (*p* < 0.05). The error bar represents the standard error of the means (n = 4).

The nutrient concentration in the grains was significantly affected (*p* < 0.05) by both the water deficit and applied Se doses (Figure 4). The nitrogen (N) concentration in the grains of plants treated with the 2.00 mg Se dm^−3^ dose decreased by 9% compared with the application of 0.25 mg Se dm^−3^ under well-watered conditions (Figure 4a). Under the water deficit, it was observed that the application of 0.50 mg Se dm^−3^ led to an average increase of 16% compared with the other doses. When comparing the irrigated and water deficit conditions, it was noted that plants subjected to water deficiency experienced a 27% increase at the 0.50 mg Se dm^−3^ dose.

Regarding the sulfur (S) concentration in the grains, it was noted that the 2.00 mg Se dm^−3^ dose decreased by 16% during the water deficit compared with the other doses. However, when comparing plants under the irrigated and water deficit conditions, it was observed that the application of 0.50 mg Se dm^−3^ resulted in a 17% increase during the deficit (Figure 4b).

The results for the total free amino acids (TFAAs) and total soluble proteins (TSPs) showed significant interactions between the Se dose and water deficit factors (*p* < 0.05). There was a 32.76% increase in TFAAs compared with the lowest Se dose (0.25 mg dm^−3^) in the irrigated condition and Se application. However, under water deficit and with the application of 0.25 mg Se dm^−3^, a ~19% increase in TFAA was observed. TSP values were higher under water deficit, with the 0.25 mg Se dm^−3^ dose rather than 2.00 mg Se dm^−3^ increasing the TSP by 57.9%. Considering the 0.00 mg Se dm^−3^ dose under irrigation and the 0.25 mg Se dm^−3^ dose, there was a 58% increase in this variable when the plants received Se supplementation and were exposed to water deficit.

**Figure 5:**
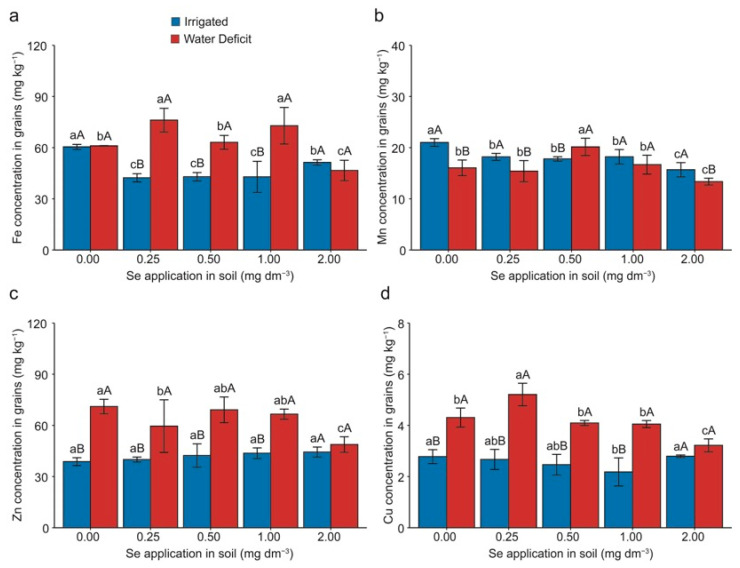
Effect of Se application and water deficit on the concentrations of iron—Fe (**a**), manganese—Mn (**b**), zinc—Zn (**c**), and copper—Cu in grains (**d**) of wheat (*Triticum aestivum* L.) plants. Lowercase letters compare the means among Se doses within each irrigation condition, and capital letters compare the irrigation conditions within each Se dose. Equal letters did not differ statistically by the Duncan test (*p* < 0.05). The error bar represents the standard error of the means (n = 4).

The Fe concentration was negatively influenced by the Se doses in plants grown under irrigation, with average decreases of 29% for the 0.25 and 0.50 mg Se dm^−3^ doses, 30% for the 1.00 mg Se dm^−3^ dose, and 16% for the 2.00 mg Se dm^−3^ dose, all compared with plants without Se. Under the water deficit, an average increase of 15% was observed for the 0.25 mg Se dm^−3^ dose compared with the 0.00, 0.50, and 1.00 mg Se dm^−3^ doses, and a 66% increase compared with the 2.00 mg Se dm^−3^ dose. When comparing irrigated plants and those under water deficit, the deficit promoted 80%, 46%, and 68% increases in the 0.25, 0.50, and 1.00 mg Se dm^−3^ doses, respectively (Figure 5a).

The Mn concentration in the grains was affected by Se application under irrigated conditions, with an average decrease of 15% for the 0.25, 0.50, and 1.00 mg Se dm^−3^ doses and a 25% decrease for the 2.00 mg Se dm^−3^ application compared with plants without Se. Under the water deficit, it was observed that the application of 0.50 mg Se dm^−3^ resulted in an average increase of 25% compared with the 0.00, 0.25, and 1.00 mg Se dm^−3^ doses and a 50% increase compared with the 2.00 mg Se dm^−3^ application. When comparing plants under irrigated and water deficit conditions, increases of 31%, 18%, and 17% were observed for the irrigated plants at 0.00, 0.25, and 2.00 mg Se dm^−3^ doses, respectively. Conversely, for the 0.50 mg Se dm^−3^ dose, a 13% increase due to water deficit was noted (Figure 5b).

Under irrigated conditions, Se application at 0.50 and 2.00 mg dm^−3^ doses promoted an average increase in the Zn concentration of 74% in the grains compared with plants without Se. However, an average reduction of 27% was noted for the 2.00 mg Se dm^−3^ application compared with the 0.00, 0.25, and 1.00 mg Se dm^−3^ doses and a 31% reduction for the 0.50 mg Se dm^−3^ dose under water deficit. Additionally, under the water deficit, the 1.00 mg Se dm^−3^ dose resulted in an 8% decrease compared with the 0.50 mg Se dm^−3^ dose. When comparing plants under the irrigated and water deficit conditions, the water deficit led to 86%, 51%, 64%, and 53% increases for the 0.00, 0.25, 0.50, and 1.00 mg Se dm^−3^ doses, respectively (Figure 5c).

For the Cu concentration in the grains, Se application at the 0.25 mg dm^−3^ dose during water deficit promoted an average increase of 25% compared with the 0.00, 0.50, and 1.00 mg Se dm^−3^ doses and a 62% increase compared with the 2.00 mg Se dm^−3^ dose. It is also noteworthy that during the water deficit, the 2.00 mg Se dm^−3^ dose resulted in an average decrease of 23% compared with the 0.00, 0.50, and 1.00 mg Se dm^−3^ doses. When comparing plants under irrigated and water deficit conditions, it was observed that the water deficit led to increases of 59%, 92%, 95%, and 95% at the 0.00, 0.25, 0.50, and 1.00 mg Se dm^−3^ doses, respectively (Figure 5d).

**Figure 6:**
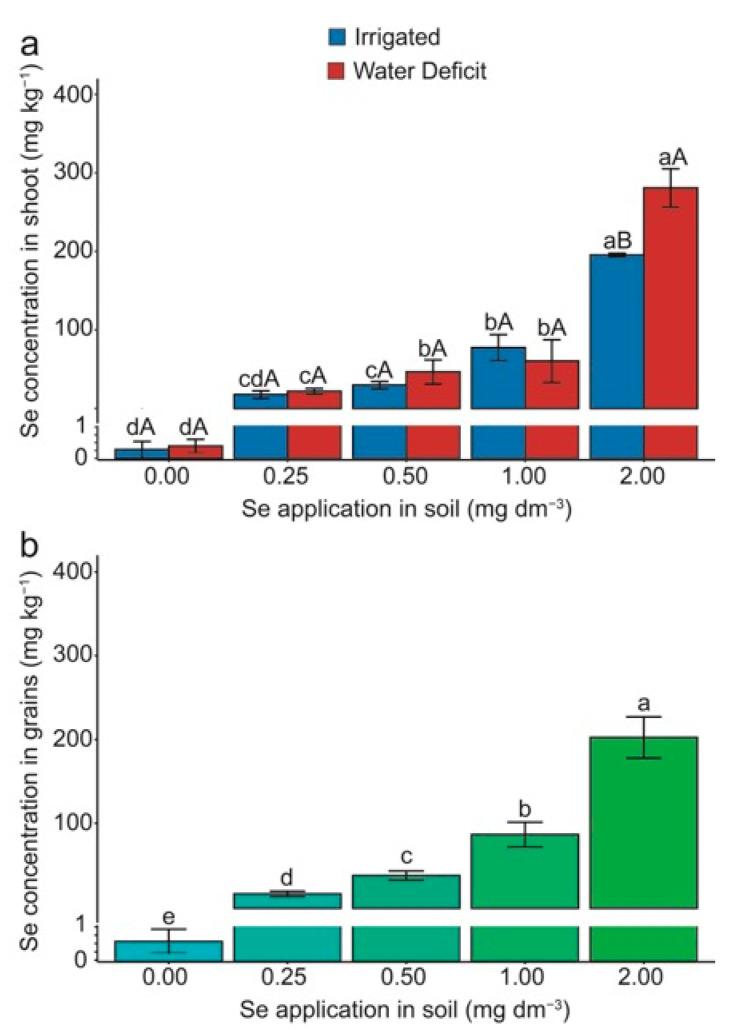
Effect of Se application and water deficit on selenium—Se concentration in the shoot (**a**) and Se concentration in the grains (**b**) in wheat (*Triticum aestivum* L.) plants. Lowercase letters compare the means among Se doses within each irrigation condition, and capital letters compare the irrigation conditions within each Se dose. Equal letters did not differ statistically by the Duncan test (*p* < 0.05). The error bar represents the standard error of the means (n = 4).

It can be noted that there was an interaction between the water regime and Se doses for the Se concentration in the shoots and grains (Figure 6). However, the Se concentration in the grains was affected solely by the Se doses. The Se concentration in the shoots increased with higher Se doses, with a notable difference at the 2.00 mg Se dm^−3^ dose, where under water deficit conditions, the Se concentration in the shoots was higher than in the irrigated condition (Figure 6a). A similar trend was observed for the Se concentration in the grains, which increased with higher Se application in the soil (Figure 6b).

**Figure 7:**
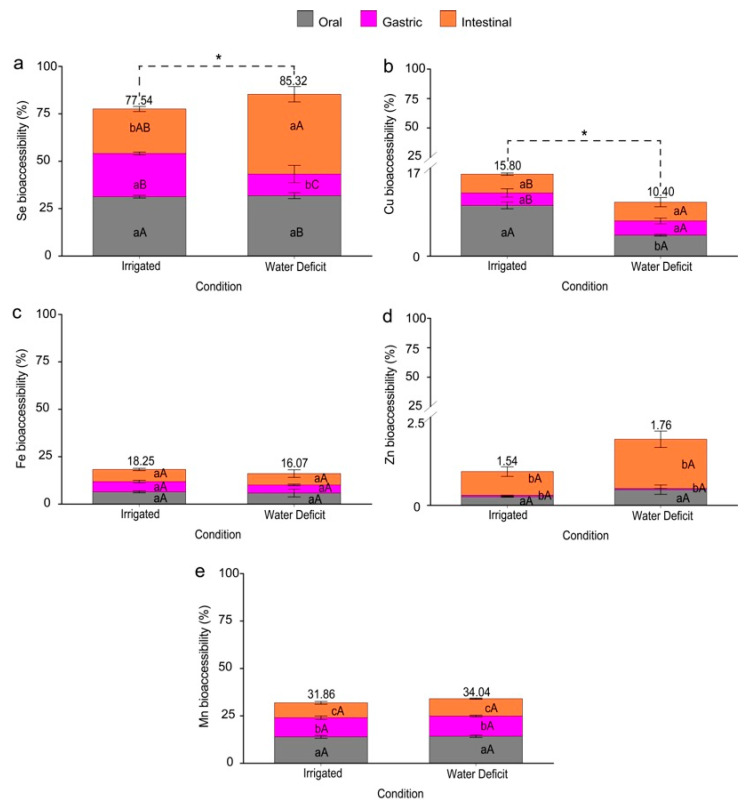
Percentage of elements’ bioaccessibility in wheat (*Triticum aestivum* L.) grain powder in the in vitro simulated human gastrointestinal system. Bioaccessibility of selenium—Se (**a**), copper—Cu (**b**), iron—Fe (**c**), zinc—Zn (**d**), and manganese—Mn (**e**). Means followed by the same capital letter within the condition and lowercase letters between conditions did not differ statistically at the 5% (*p* < 0.05) probability level by the Duncan test. The error bars represent the standard error of the means (n = 4). Asterisks (*) refer to the significant difference regarding the total bioaccessibility of the elements when comparing the conditions.

The irrigated condition influenced the total bioaccessibility of Se and Cu (Figure 7a,b). When plants were subjected to water deficit, there was a 7.78% increase in the total Se bioaccessibility (Figure 7a). A greater contribution from the intestinal phase was observed under water deficit, which was higher than the other phases compared to the irrigated condition. Only the gastric phase differed from the water deficit under the irrigated condition. There was a 5.4% increase for Cu when the plants were under irrigation (Figure 7b). However, only the oral phase was lower under the water deficit than the irrigated condition.

For the elements Fe, Zn, and Mn (Figure 7c–e), there was no difference in the total bioaccessibility values, and the comparison between the irrigation management phases also showed no statistical difference. Only for Mn (Figure 7e) was there a difference between the phases; when the plants were irrigated, the oral phase showed greater bioaccessibility, followed by the gastric and intestinal phases, a similar behavior observed in the plants under water deficit.

### 2.5. Multivariate Analysis

**Figure 8:**
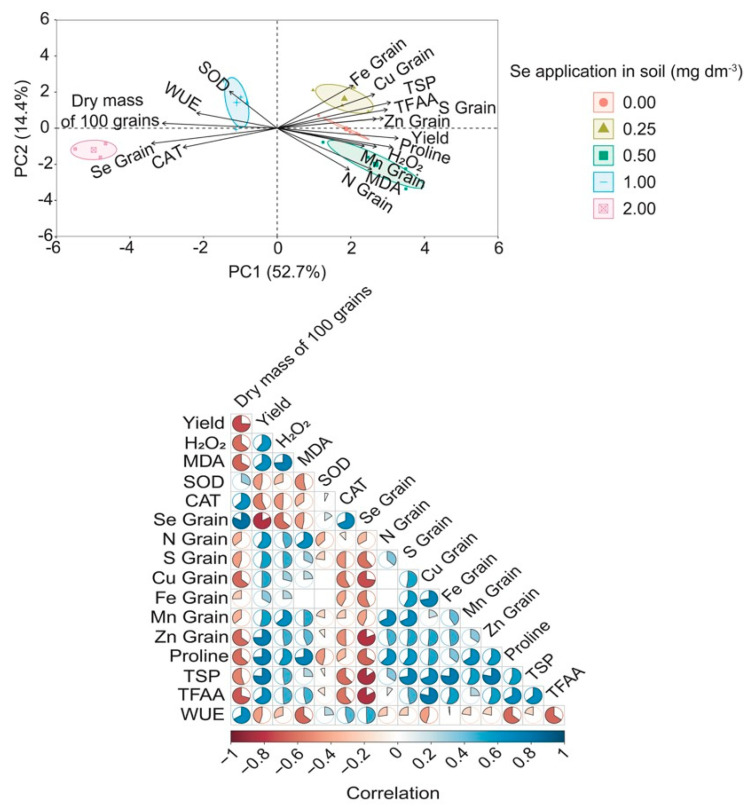
Principal component analysis and Pearson of physiological, metabolic, and nutritional responses in the wheat (*Triticum aestivum* L.) leaves and grains cultivated with different Se under water deficit. In leaves: CAT—catalase; SOD—superoxide dismutase; MDA—malondialdehyde; H_2_O_2_—hydrogen peroxide; WUE—water use efficiency; and proline. In grains: TSPs—total soluble proteins; TFAAs—total free amino acids; NG—nitrogen; SG—sulfur; FeG—iron; CuG—copper; ZnG—zinc; MnG—manganese; SeG—selenium. Significant correlation coefficients (*p* < 0.005) are indicated by colored circles where negatives are closer to black and positives to blue, respectively. Blank spaces indicate non-significance.

Principal component analysis (PCA) and Pearson correlation for wheat plants under water deficit (Figure 8) revealed that PCA1 and PCA2 accounted for 67.1% of the total data variance. Supplementation with 0.25 mg Se dm^−3^ improved the grain quality by increasing the concentrations of Fe, Cu, S, Zn, TFAAs, and TSPs and showed a tendency to enhance the grain yield. An inverse relationship was observed between the grain quality, Se concentration, and CAT activity at the 2.00 mg Se dm^−3^ dose. This result suggests that high Se doses can impair the grain quality and yield, even when the CAT activity is increased and oxidative stress is reduced (Figure 3a,b).

This data pattern can also be explained by the hormesis effect, where appropriate Se concentrations (0.25 mg dm^−3^) benefit wheat plants under water deficit, enhancing the grain quality. Conversely, a high Se dose (2.00 mg dm^−3^) has the opposite effect, suggesting that under stress conditions, Se is beneficial when provided within an optimal range.

The 0.25 mg Se dm^−3^ dose favored most variables under severe water deficit. However, Pearson correlation revealed that the H_2_O_2_, and MDA concentrations positively correlated with the grain yield, with coefficients of 0.60 and 0.65, respectively (Figure 8). Therefore, we can presume that the low H_2_O_2_ concentration found in this study (Figure 3a,b) may act as a signaling molecule in wheat cells for detecting water stress, potentially contributing to the adjustment of the plant’s defense mechanisms.

## 3. Discussion

### 3.1. Grain Productivity and Mass

One of the significant challenges in cereal cultivation under drought conditions is the induced abortion of flowers and grains [19], leading to drastic yield losses. Our results demonstrate that severe water deficit causes significant productivity losses and that increasing Se doses directly affects the grain yield (Figure 1a). However, the literature reports that adequate Se doses can enhance production or reduce losses in various crops such as barley [20], wheat [21,22], maize [23], cauliflower [24], and okra [25].

In our study, plants under low Se doses experienced fewer yield losses than those under higher Se doses during water deficit (Figure 1a). Conversely, higher Se doses increased the mass of 100 grains (Figure 1b). These results suggest that the production of heavier grains is likely due to a concentration effect, where the reduced grain number results in increased mass, albeit with substantial yield losses.

Our findings indicate that productivity is inversely proportional to grain mass; as the Se doses increased, the productivity decreased while the grain mass increased. This situation suggests a direct relationship between yield and grain mass (Figure 1a,b). These results are similar to those found by [21], who studied wheat plants under water stress and exogenous Se addition. However, the authors attributed their results to the effects of Se on plant water relations, osmoprotectants, and increased antioxidant activity.

### 3.2. Gas Exchange

Applying Se under optimal growing conditions did not significantly affect the CO_2_ assimilation rate (Figure 2). The reduction in photosynthetic rate under water stress, compared with the optimal conditions (Figure 2a), is directly related to chlorophyll degradation, stomatal closure, suppression, and fragmentation of RuBisCO (ribulose-1,5-bisphosphate carboxylase-oxygenase, EC 4.1.1.39), leading to a decreased photochemical rate of photosystem II [26,27]. This situation likely resulted in substantial losses in CO_2_ assimilation by the plants. The literature shows that Se can mitigate damage to chloroplast structures and photosystem II reaction centers in plants under water stress [15,28]. However, due to the phenological stage of the crop and the induced water stress, this positive effect of Se might not have been observed. Under water deficit at doses of 0, 0.25, and 0.50 mg Se dm^−3^ (Figure 2a), the plants exhibited negative photosynthesis, meaning that instead of assimilating CO₂, they began releasing it, indicating elevated photorespiration. In this situation, respiration instead of photosynthesis can be one of the main factors contributing to yield loss [29]. However, this effect was not observed at the 0.50 mg Se dm^−3^ dose (Figure 1a) in the present study, which can be explained by the higher CAT activity (Figure 3d). This enzyme showed increased activity under higher H₂O₂ concentrations (Figure 3a) as well as a higher concentration of osmoprotectants like proline (Table 1).

The higher WUE by plants at higher Se doses under water stress (Figure 2b) could be due to the photosynthetic rate with transpiration adjustment. Thus, applying Se in wheat plants under water deficit can increase the water retention and maintain the transpiration rates. The authors in [30] suggest that the protective effect of Se on plants under water stress is related to the active absorption of water from the soil by the roots rather than the loss of water through transpiration.

### 3.3. Enzymatic Antioxidant Metabolism and Oxidative Damage

The H_2_O_2_ concentrations in plants treated with Se and subjected to induced water stress (Figure 3a) were lower than those proposed by [31] in non-stressed plants. This result indicates that the application of Se allows plants to maintain lower H_2_O_2_ levels even under harmful conditions, which aligns with the higher CAT activity (Figure 3d). This enzyme catalyzed the conversion of H_2_O_2_ into H_2_O and O_2_ [32] compared with the treatment without Se addition.

Lipid peroxidation, estimated through MDA, was reduced as Se doses increased in plants under water stress (Figure 3b). This situation occurs because Se compounds act directly or indirectly on the scavenging of reactive oxygen species (ROS) [14]. Like the present study, Hasanuzzaman et al. [33] observed a decrease in MDA in canola seedlings when applying Se under water stress. It is very important to keep ROS at a proper level because these molecules can lead to cell death by causing oxidative damage, decreasing the plasma membrane fluidity and damaging membrane proteins, ion channels, and enzymes [34,35].

The proline concentration in wheat plants was higher when the plants received the lowest Se doses, both with and without water deficit, and decreased at higher Se doses (Table 1). This result could be related to a pro-oxidant effect of Se at higher doses. Under stress conditions, higher proline concentrations can benefit plants, with its free accumulation being an adaptation induced by water and salt stress [36]. Proline production under severe water deficit may be an adaptive trend for plants to survive stress. This condition might be related to the chelating ability of this amino acid to sequester metal ions, which can be used as a defense mechanism in stressed plants [37]. In wheat seedlings, increased proline concentration improved the plant conditions under water deficit due to Se application as it improved the water status of the plants [38].

### 3.4. Grain Quality and Bioaccessibility in the Simulated Human Gastrointestinal System

Our results demonstrate that water stress increased the concentrations of N and S in the grains compared with the treatments without water deficit (Figure 4a,b). Research suggests that regulating and redistributing essential elements such as N and S by Se is associated with the reactivation of the antioxidant system to reduce ROS and improve tolerance to abiotic stress [39]. However, the effects of Se on the regulation of essential elements in stressed plants are not yet fully understood. Se (21 and 38 g ha^−1^) has been shown to increase the N concentration in wheat grains [40]. In wheat plants, the regulation of N metabolism is associated with increased nitrate reductase (EC 1.6.6.1) enzyme activity [41]. Thus, Se positively influences amino acid metabolism in plants, promoting greater N uptake and assimilation [42].

The biosynthesis and accumulation of amino acids are triggered by environmental stresses including water stress [43]. This results in the breakdown of structural proteins into amino acids, which participate in osmotic adjustment under water stress conditions [44,45]. A higher accumulation of amino acids and proteins in the grains is associated with a higher rate of N assimilation. Our results are interesting because, despite severe stress, as applied in our wheat study, producing grains richer in amino acids and proteins (Figure 4c,d) is promising for more balanced and nutritious human diets.

Elements such as Fe, Mn, Zn, and Cu are essential for SOD activity, as they are its cofactors. SOD is the first enzymatic antioxidant barrier, catalyzing the dismutation of O^2−^ into H_2_O_2_ [46,47]. Under stress conditions, plants tend to increase the concentration of essential elements to enhance the activity of antioxidant enzymes [48]. However, a higher concentration of these micronutrients in the grains benefits the quality of the harvested product. Except for Se, which increased in concentration in the grains with higher Se doses (Figure 6b), and Mn, which had higher concentrations at an intermediate Se dose (Figure 5b), other elements (Fe, Zn, and Cu) showed significant increases under water deficit at the lowest Se dose (Figure 5a,c,d). These results are encouraging because the grains became more nutritionally rich, even with severe water stress.

Foliar application of selenate increased the Fe concentrations in rice grains [49]. However, this significant effect differs from observations in rice plants under water stress [50]. Se supplementation showed Fe accumulation in wheat plants [48]. Tobiasz et al. [51] found decreasing Mn levels at higher Se doses in wheat plants. Similarly, lettuce plants showed a 36% reduction in Mn concentration under increasing Se doses [52]. The Zn concentration was significantly higher in grains under water stress, and a higher Zn concentration in the shoots and grains of wheat associated with environmental stress has been reported [53].

In contrast, [21] observed a 42% decrease in Zn concentration in wheat grains under water deficit, although they applied mild stress compared with the severe stress in this study. Andrade et al. [50] noted that Se application linearly increased the Cu concentration in rice regardless of the water regime. Arvy et al. [54] also reported an increased Cu concentration in *Catharanthus roseus* (L.) biomass with Se application. Despite its low mobility, Cu is required in low concentrations by plants and can be translocated from vegetative organs to grains, especially pronounced under Se and water stress conditions. The authors in [45] found higher Cu concentrations in rice grains with Se supplementation, suggesting a possible synergistic effect between Se and Cu.

Roots absorb selenate via sulfate transporters, although plants can exhibit different selectivities between sulfate and selenate [11,55]. Plants assimilate inorganic forms of Se into SeCys and SeMet through sulfur transporters [56]. Thus, we can assume that a higher concentration of sulfur in the grains under lower doses of Se and water deficit (Figure 4b) may be related to a lower incorporation of seleno-amino acids (SeCys and SeMet) through the substitution of Cys and Met in the protein chain. The translocation of Se in plants supplemented with selenate quickly occurs from the root xylem to shoot tissues [52]. Guerrero et al. [42] observed that wheat seedlings grown in a hydroponic system with selenate accumulated large amounts of Se. These results were similar when using 20 μM Se L^−1^ in different wheat lines, with accumulations of 400 to 600 mg Se kg^−1^ [52]. Although wheat is not considered a Se accumulator [57], it has a relatively high tolerance compared with other species [39,58].

Figure 6a shows that increasing doses of sodium selenate associated with water stress significantly increased the Se concentration in the shoot. Although it increased linearly, Se concentration in the grains did not show a significant effect between doses and water stress imposition (Figure 6b). Boldrin et al. [57] observed a linear increase in Se concentration in the shoot of wheat seedlings. In maize under water deficit, Bocchini et al. [23] linked the ability to accumulate high Se concentrations in tissues to the plants’ capacity to divert Se from selenocysteine (SeCys) and selenomethionine (SeMet) to methyl selenocysteine (MeSeCys). Wheat plants’ remarkable ability to translocate and store selenate in the shoots is related to two complementary mechanisms. Initially, selenate is accumulated in the cell vacuole as a protective measure against toxicity. Subsequently, Se can be eliminated through volatile compounds [42]. Se compartmentalization in the vacuole also reduces its mobilization and incorporation into the chain protein [59].

Considering the bioaccessibility of Se (Figure 7a) and the daily consumption of wheat (~180 g day^−1^), the values for the reference daily intake of this element are well above the recommended levels [60]. However, the study was conducted in a greenhouse, and the doses used were much higher than those in the field. The bioaccessible values of Se were more significant when the plants were grown under water restriction, with the intestinal phase showing the highest percentage of bioaccessibility. This condition occurs because the intestine represents the largest proportion of bioaccessibility in the human gastrointestinal system [61].

Micronutrients such as Cu have a greater affinity for low molecular weight organic compounds such as amino acids than elements like Zn and Mn [62,63]. This affinity increases Cu’s chelation, likely contributing to its higher bioaccessibility in the gastrointestinal system than Zn. This fact is widely accepted, since the two main factors affecting Zn absorption in adult diets are the relatively low concentrations of Zn in flour and phytate in the human diet [64,65]. do Nascimento et al. [66] assessed the bioaccessibility of cereal flours, such as oats, rice, and wheat, using in vitro gastrointestinal digestion. In all the flour-based foods analyzed, the bioaccessibility of Zn was low, while that of Cu exceeded 50%. These results differ from those found in the present study, where the bioaccessibility of Zn was 1.54% and 1.76% (Figure 7d), and Cu was 15.8% and 10.4% (Figure 7b) under the irrigated and water deficit conditions, respectively.

Regarding the cereal flour samples mentioned earlier, the bioaccessibility of Mn was below 50%, with values similar to those found in this study: 31.86% and 34.04% under the irrigated and water deficit conditions, respectively (Figure 7e). Two samples composed of oat and rice flour as well as whole wheat flour showed bioaccessible fractions of Fe below 35%, which also corroborates our data, where the bioaccessibility of Fe was 18.25% and 16.07% under the irrigated and water deficit conditions, respectively (Figure 7c).

It is essential to highlight that bioaccessibility analyses refine biofortification studies; in vitro simulation may not establish all of the control mechanisms for the absorption of elements and their subsequent utilization in metabolism. In vitro experiments often do not consider the specific conditions of each individual such as the concomitant intake of other foods [67].

## 4. Materials and Methods

### 4.1. The Experimental Design

**Figure 9:**
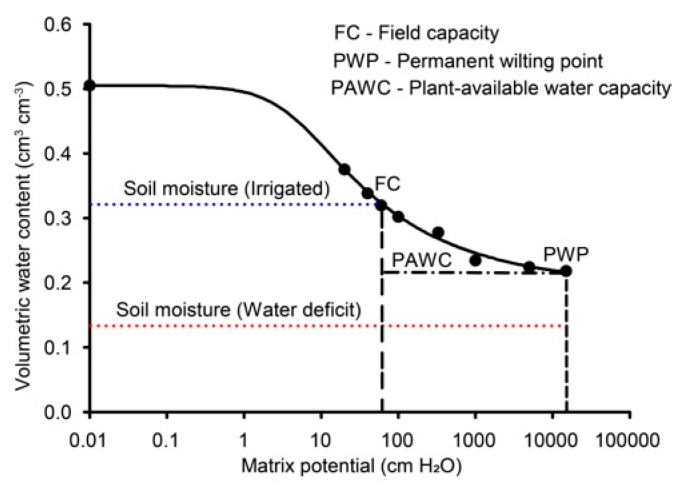
Soil water retention curve used in the experiment. The blue line represents the soil moisture in conditions without water deficit, and the red line represents the soil moisture on the last day of water restriction.

The experiment was conducted in a greenhouse (21°13′34″ S 44°58′44″ W) at the Department of Soil Science, Federal University of Lavras, Minas Gerais, Brazil. The experimental design was completely randomized in a 5 × 2 factorial scheme with four replications. The first factor consisted of five doses of Se (0, 0.25, 0.50, 1.0, and 2.0 mg dm^−3^) applied to the soil in the form of sodium selenate (Na_2_SeO_4_), and the second factor involved two irrigation conditions (with and without water deficit). The water deficit was imposed during the grain-filling phenological stage (stage 7.0 of the Zadoks scale, [68]) when at least 80% of the plants had completed anthesis (59 days after emergence). Plants under water deficit were maintained until the soil matric potential exceeded the permanent wilting point (−1500 kPa or 15,000 cm H_2_O), which was considered a severe water deficit. Water deficit monitoring was carried out according to [69]; additionally, a water retention curve was established to determine the water content at field capacity (−6 kPa or 60 cm H_2_O) and at the permanent wilting point (−1500 kPa or 15,000 cm H_2_O) (Figure 9).

The soil used in the experiment was classified as Latossolo Vermelho Distroférrico according to the Brazilian Soil Classification System [70] and Hapludox according to the U.S. Soil Taxonomy System [71]. It was collected from a native area and analyzed following the methodology of [72] (Table S1 in the Supplementary Materials). The soil was amended to raise its base saturation to 60% [73]. Fertilization was carried out according to the recommendations for pot experiments proposed by [74]. Thus, the following nutrients (in mg dm^−3^) were added to the pots: 300 N (ammonium nitrate), 200 phosphorus P (monoammonium phosphate), 150 potassium K (potassium chloride), 50 S (ammonium sulfate), 0.5 boron B (boric acid), 1.5 Cu (copper sulfate), 0.1 molybdenum Mo (ammonium molybdate), and 5.0 Zn (zinc sulfate).

### 4.2. Biometric Analysis of Plants

At the end of the experiment, the grains were harvested, stored in paper bags, and dried in a forced air circulation oven until reaching a constant weight. The moisture content was then adjusted to 13% [75], and the grains were weighed to determine the grain yield per pot. Additionally, 100 grains were randomly selected to determine the mass of 100 grains.

### 4.3. Gas Exchange

The analyses were conducted on the sixth day of the water deficit. A portable infrared gas analyzer (IRGA, model LICOR 6400, Li-CORBiosciences, Lincoln, NE, USA) was used for this purpose. Data were collected between 8 and 10 a.m., evaluating the following variables: photosynthetic rate (*A*—μmol CO_2_ m^–2^ s^−1^) and transpiration rate (*E*—mmol H_2_O m^−2^ s^−1^). Based on the results of the photosynthetic rate and transpiration rate, the WUE was estimated [WUE = A/E (μmol CO_2_ mmol^−1^ air^−1^)]. The atmospheric CO_2_ concentration inside the leaf chamber was maintained at 400 μmol CO_2_ and irradiance at 1500 μmol m^−2^ s^−1^. The minimum pre-established stabilization time for the readings was 120 s.

### 4.4. Biochemical Analysis

#### 4.4.1. Hydrogen Peroxide and Malondialdehyde

In assessing H_2_O_2_ and lipid peroxidation, 0.2 g of fresh material was collected and ground in liquid nitrogen, homogenized in 1500 μL of trichloroacetic acid, and centrifuged at 12,000× *g* for 15 min at 4 °C. The hydrogen peroxide content was determined by collecting the supernatant and then reading the absorbance at 390 nm in a 10 mM potassium phosphate buffer (pH 7.0) with 45 μL of the plant material extract and 1 M potassium iodide [76].

Lipid peroxidation was quantified following the methodology of [77]. For this, 125 μL of the extraction supernatant was collected and pipetted into a 1500 μL microtube containing 250 μL of the following reaction medium: 0.5% thiobarbituric acid (TBA) and 10% trichloroacetic acid (TCA). Subsequently, the microtubes were placed in a water bath at 95 °C for 30 min, and after that, the reaction was stopped in a container containing ice. Then, 350 μL of the reaction medium was collected and pipetted into microplates, and the absorbance was read on a spectrophotometer at 535 nm and 600 nm. The quantification of MDA was performed according to Equation (1):

[MDA] = (A535 − A600)/(ξ.b) (1)

where ξ is the molar extinction coefficient = 1.56 × 10^−5^ cm^−1^) and b is the optical path length = 1). Lipid peroxidation was expressed in nmol (MDA) g^−1^ of fresh weight.

#### 4.4.2. Antioxidant System Enzymes

For the enzymatic evaluation of SOD and CAT, 0.2 g of fresh material was collected and underwent grinding in liquid nitrogen, followed by the addition of 1.5 mL of a buffered solution (0.1 mol L^−1^ potassium phosphate pH = 7.8, 0.1 mol L^−1^ EDTA pH = 7.0, 0.5 mol L^−1^ DTT, 0.1 mol L^−1^ PMSF, 1 mmol L^−1^ ascorbic acid, and 22 mg of PVPP) [78]. Subsequently, the suspension was centrifuged at 13,000× *g* for 10 min at 4 °C, and the supernatant was collected for analysis using a spectrophotometer (Epoch BioTek ELISA Plate Reader), manufactured by BioTek Instruments, Inc., based in Winooski, VT, USA. Following the methodologies of [79,80] for SOD and CAT, respectively.

#### 4.4.3. Total Free Amino Acids, Proline, and Total Soluble Proteins

For the determination of total free amino acid content, a reaction medium was prepared containing (A) 0.2 M sodium citrate buffer (pH 5.0); (B) 5% ninhydrin in Methyl Cellosolve (ethylene glycol monomethyl ether); (C) 2% KCN in Methyl Cellosolve (2 mL of 0.01 M KCN in 100 mL of Methyl Cellosolve) and 60% ethanol (*v*/*v*). In microtubes, 1 mL of the extract, (A) 0.5 mL, (B) 0.2 mL, and (C) 1 mL, and 1 mL of distilled water were added. Subsequently, the tubes were placed in a water bath for 20 min at 100 °C. After cooling to room temperature, 1.3 mL of ethanol was added, and quantification was performed by collecting 350 μL of the reaction medium, placed in microplates, and read at 570 nm [81]. The total free amino acid content was expressed in μmol g^−1^ of dry mass.

Extraction and quantification of the proline contents followed the methodology proposed by [82]. Briefly, 0.2 g of plant material was mixed with 10 mL of 3% sulfosalicylic acid and stirred for 60 min at room temperature. After filtration, the tubes were placed in the water bath at 100 °C for 60 min. The tubes were cooled on ice, and the reading was performed using a microplate spectrophotometer at 520 nm. Quantification was performed using a standard analytical curve of proline.

The total soluble proteins and free amino acids were extracted using an ethanol extraction method adapted from [83]. Firstly, ethanol extractions were performed sequentially using 100%, 80%, and 50% ethanol. For the protein content determination, 6 μL of the supernatant was added to microplates, pre-filled with 294 μL of Bradford solution (1:5 reagent dilution). Absorbance readings were taken using a microplate reader (Epoch-BioTek-Elisa) at a wavelength of 595 nm, and the results were obtained from a calibration curve expressed in mg g^−1^.

### 4.5. Analysis of the Nutritional Status of Plants

For the determination of Se concentrations in the shoot and grains as well as S, Cu, Fe, Mn, and Zn in the grains, the plant materials underwent acid digestion using protocol 3051A from the United States Environmental Protection Agency—U.S. EPA [84]. The protocol involves digestion in concentrated nitric acid (HNO_3_) using a CEM^®^ Mars-5 microwave system (CEM Corp, Matthews, NC) with pressure and temperature control.

The plant material, leaves, and grains were dried in a forced air circulation oven and ground using a Willey mill. For digestion, 0.5 g of plant material was mixed with 5 mL of distilled nitric acid (HNO_3_).

The determination of S, Cu, Fe, Mn, and Zn concentrations in the extract was conducted using inductively coupled plasma-optical emission spectrometry (ICP-OES) (Spectro, model Blue, Germany) with background correction. The concentration of N was determined by digesting 0.1 g of plant material in 3 mL of sulfuric acid (H_2_SO_4_), followed by distillation of the samples using the Kjeldahl method [85].

The evaluation of Se concentrations in the digested solutions was carried out using inductively coupled plasma-mass spectrometry (ICP-MS). During the analyses, certified samples of plant material were employed in quality control and assurance protocols (QA/QC protocol), with a Se recovery rate of 80.50% ± 0.02% (n = 4). A certified reference material from the Institute for Reference Materials and Measurements (White Clover—BCR 402, IRMM, Geel, Belgium) was used, and a blank sample was added for each set of analyses to ensure the reliability of the results.

### 4.6. Bioaccessibility in the Simulated Human Gastrointestinal System

For in vitro bioaccessibility, the method [86] proposed was used, with a modification in the oral phase by adding the enzyme α-amylase. This modification was chosen due to the high starch concentration in wheat grains. The simulation of the oral phase consisted of a solution of monobasic potassium phosphate (KH₂PO₄), sodium hydroxide (NaOH), and the enzyme α-amylase (activity of 75 U min^−1^) with the pH adjusted to 6.8. The gastric phase was simulated using a solution of pepsin, sodium chloride (NaCl), and distilled hydrochloric acid (HCl), with the solution pH set at 1.8. For the intestinal phase, a solution with pH = 6.8 was prepared using pancreatin, monobasic potassium phosphate (KH₂PO₄), and sodium hydroxide (NaOH).

The in vitro bioaccessibility assay began with the simulation of the oral phase, in which 0.5 g of grain powder was mixed with 3 mL of simulated saliva in 15 mL tubes. These tubes were placed in a water bath at 37 °C for 10 min to simulate human physiological conditions. Subsequently, the tubes were centrifuged at 1915× *g* for 15 min, and the supernatant was collected. Next, 3 mL of the gastric solution was added to the pellet, and the tubes were placed in a water bath at 37 °C for 2 h. As in the previous step, the tubes were centrifuged again, and the supernatant was separated into another tube. The intestinal phase was performed by adding 3 mL of the intestinal solution, following the same steps as the previous phase. The residual fraction of Fe, Mn, Zn, Cu, and Se was determined from the residual pellet of the final (intestinal) phase. The solid material was dried and digested to determine the non-bioaccessible levels.

The supernatant was digested with 1 mL of concentrated nitric acid (HNO₃), and 2 mL of H₂O₂ was added to the solutions to reduce the organic carbon in the samples to determine the bioaccessible levels at each phase, simulating the gastrointestinal system [87]. Subsequently, the percentage of bioaccessible fractions was calculated for each phase.

### 4.7. Statistical Analyzes

The data were submitted to the normality test (Shapiro–Wilk) and homogeneity of variance (Bartlett). The means were submitted for the Duncan test, and the results presented as bar graphs. The R software, version 4.4.2 [88]) was used for statistical analysis and plotting the graphs as well with the packages ggplot2 [89], FactoMineR [90], factoextra [91], tidyverse [92], Factoshiny [93], multcomp [94], and dplyr [95].

## 5. Conclusions

This study identified the effects of Se application under water stress conditions in wheat cultivation. It was observed that soil-applied Se in sodium selenate progressively increased the Se concentration in the wheat grains. This effect is significant because grains are the consumable part of the plants for humans and animals. Under stress conditions, Se at a dose of 0.25 mg dm^3^ was beneficial by allowing lower yield losses while enriching the grains with Se. This positive effect of Se was also evident under oxidative stress, where Se increased the CAT activity, reducing H_2_O_2_ and lipid peroxidation. Furthermore, Se application increased the grain mass and production of nutritionally richer grains, with higher concentrations of N, S, total free amino acids, total soluble proteins, Fe, Zn, and Cu. In addition, the association of Se with water deficit increased the bioaccessibility of this element in the simulated gastrointestinal system, contributing to food security. Since this study was conducted in a greenhouse, field experiments are recommended to better define the optimal Se dose for mitigating water stress in wheat plants and to determine the appropriate Se concentration in wheat grains for human consumption.

## Figures and Tables

**Table 1:** Effect of Se application and water deficit on proline in wheat (*Triticum aestivum* L.) plants.

Condition	Se Application in Soil (mg dm^−3^)				
	0.00	0.25	0.50	1.00	2.00
Irrigated	202.24 ± 9.31 bA	285.98 ± 5.52 aA	253.96 ± 6.59 aB	194.26 ± 20.91 bA	68.10 ± 1.42 cA
Water Deficit	207.96 ± 19.10 cA	280.25 ± 10.11 bA	367.10 ± 44.42 aA	123.42 ± 6.93 dB	69.37 ± 3.85 eA
*p*-value	0.007647 *				
CV (%)	17.16				

Means followed by the same uppercase letter in the column and lowercase letters in the row did not differ statistically (*p* < 0.05) by the Duncan test. Error bars represent the standard error of the means (n = 4). The asterisk (*) indicates that the result is statistically significant at the 0.05 level.

## Data Availability

All data generated or analyzed during this study are included in this published article and its Supplementary Materials. However, the raw dataset can be requested from the corresponding author on reasonable request.

## Supplementary Materials

The following supporting information can be downloaded at https://www.mdpi.com/article/10.3390/plants13243460/s1, Table S1: Physical and chemical composition of the soil (0.00–0.20 m used in the experiment.
