# Supplementary material for: Soil Application of Selenium in Wheat (Triticum aestivum L.) Under Water Stress Improves Grain Quality and Reduces Production Losses
Source: Plants (Basel). 2024 Dec 10;13(24):3460. doi: 10.3390/plants13243460 (PMC11677958; doi:10.3390/plants13243460)
Supplement: Supplementary file 1 [file plants-13-03460-s001.zip › plants-3315370-supplementary.pdf]

Table S1. Physical and chemical properties of the soil (0.00-0.20 m) used in the experiment.

Soil properties	Unit of measurement	Value
pH (H_2O)	-	4.60
SOM	g kg^{-1}	32.7
P (Mehlich-I)	mg dm^{-3}	1.18
Fe^{2+}	mg dm^{-3}	171.29
K^+	mg dm^{-3}	61.90
Ca^{2+}	$cmol_c$ dm^{-3}	0.45
Mg^{2+}	$cmol_c$ dm^{-3}	0.28
Al^{3+}	$cmol_c$ dm^{-3}	1.18
H+Al	$cmol_c$ dm^{-3}	11.62
P - rem	mg L^{-1}	18.34
Se	mg kg^{-1}	0.27
Sand	g kg^{-1}	280
Silt	g kg^{-1}	110
Clay	g kg^{-1}	610

pH was determined in water at a ratio of 1:2.5 (w/v). Soil organic matter (SOM) was determined by the Walkley-Black method. Clay, silt, and sand were assessed using the Boyoucos method. The available contents of K, P and Fe were determined by the Mehlich-1 soil test. Ca^{2+} and Mg^{2+} exchangeable contents were extracted by a 1 mol L^{-1} KCl solution-soil test.
